# HIV/AIDS, conflict and security in Africa: rethinking relationships

**DOI:** 10.1186/1758-2652-11-3

**Published:** 2008-09-22

**Authors:** Joseph U Becker, Christian Theodosis, Rick Kulkarni

**Affiliations:** 1 Section of Emergency Medicine, Department of Surgery, Yale University School of Medicine, 464 Congress Avenue, Suite #260, New Haven, CT 06519, USA; 2 Emergency Medicine, University of Chicago, 5841 S. Maryland Avenue, Chicago, IL 60638, USA; 3 Medical Director, Adult Emergency Department, Yale-New Haven Hospital, Assistant Professor of Surgery, Section of Emergency Medicine, Department of Surgery, Yale University School of Medicine, 464 Congress Avenue, Suite #260, New Haven, CT 06519, USA

## Abstract

The effect of conflict on HIV transmission and regional and global security has been the subject of much recent discussion and debate. Many long held assumptions regarding these relationships are being reconsidered. Conflict has long been assumed to contribute significantly to the spread of HIV infection. However, new research is casting doubt on this assumption. Studies from Africa suggest that conflict does not necessarily predispose to HIV transmission and indeed, there is evidence to suggest that recovery in the "post-conflict" state is potentially dangerous from the standpoint of HIV transmission. As well, refugee populations have been previously considered as highly infected vectors of HIV transmission. But in light of new investigation this belief is also being reconsidered. There has additionally been concern that high rates of HIV infection among many of the militaries of sub-Saharan Africa poses a threat to regional security. However, data is lacking on both dramatically elevated prevalence amongst soldiers and a possible negative effect on regional security. Nevertheless, HIV/AIDS remain a serious threat to population health and economic well being in this region. These issues are of vital importance for HIV programming and health sector development in conflict and "post-conflict" societies and will constitute formidable challenges to the international community. Further research is required to better inform the discussion of HIV, conflict, and security in sub-Saharan Africa.

## Introduction

HIV and AIDS pose serious threats to global health. While efforts to address the epidemic have been complicated by innumerable social, cultural and economic factors, one factor, that of conflict, and the societal disarray that often follows, creates a unique environment potentially conducive to epidemic spread. Indeed, poverty, interrupted access to health resources, stress, and poor nutritional support are commonly associated with conflict or post-conflict zones. The past two decades have witnessed a multitude of conflicts and wars in regions of poor baseline health and relatively high HIV prevalence. Sub-Saharan Africa in particular, has witnessed multiple conflicts both within and across national borders. Conflicts in this region have created widespread population displacement. Individuals deprived of their home social and economic networks frequently engage in high-risk behaviors increasing their vulnerability to HIV infection [1-4]. Despite this, recent data suggests that conflict and population displacement may not automatically equate elevated HIV prevalence [5,6]. Likewise, recovery and reconstruction may not necessarily lead to improvements in health and well being, as the distinction between conflict and "post-conflict" states is often artificial. Indeed, the "post-conflict" period is often associated with persistent deterioration of law and order, surpluses of arms and unemployed former combatants as well as continued interruption of social and health infrastructure. As HIV and conflict continue to menace poorly resourced nations, there is concern that the impact of these two factors will impact regional and global security. However, no firm data exists demonstrating this effect. As such, previously held assumptions regarding HIV, conflict, recovery and their impact on security have undergone recent examination and reconsideration.

In this document we review the recent data regarding the HIV epidemic in populations affected by conflict in sub-Saharan Africa. Further, we discuss recent discourse in relation to the effect of HIV on security. Future directions and avenues for intervention are examined with particular attention paid to the issues facing nations emerging from conflict.

### Epidemiology of HIV/AIDS in Conflict

It has previously been considered evident that conflict aids the potential transmission of HIV through the disruption of protective social and family networks as well as the interruption of vital social and health services [2-4]. It is also known that populations living in conflict zones are vulnerable to sexual violence, malnutrition, and substance abuse. All of these are risk factors for HIV transmission or the development of AIDS [1-4]. However, recent work suggests that the relationship between HIV and conflict may not be straightforward. During the last decade several African conflict zones have demonstrated lower than expected HIV prevalence. Sierra Leone, after decades of conflict had an HIV prevalence of only 0.9% in 2002 [5]. This was not appreciably higher than estimates from years earlier in the conflict and was lower than many neighboring countries not involved in conflict, including Guinea, where HIV prevalence ranged from 2.1 to 3.7%, depending on region. [4-6]. The same trend is notable in Southern Sudan where conflict between pro-government militias and local rebel groups continues. HIV prevalence has not climbed appreciably even after several years of conflict and remains low in comparison to neighboring countries [6,7]. The explanation for these findings is unclear, as these conflicts have unfortunately been rife with sexual violence, population displacement and disruptions of health and social infrastructure.

Other examples point towards a positive correlation between conflict and HIV infection. The conflict between Tanzania and Uganda in the 1970s is thought to have contributed significantly to the spread of HIV in these two countries [8]. Retrospectively, researchers have suggested that occupation of communities in both these countries by military forces as well as commercial sex work were at least partially to blame for the increases in HIV prevalence [8].

The interplay of conflict and HIV prevalence was addressed in a systematic fashion in a recent study by Spiegel et al [6]. The authors examined HIV prevalence data from seven separate African conflict zones. Conflict countries included in the study were Rwanda, Democratic Republic of the Congo, Burundi, Uganda, Southern Sudan, Sierra Leone and Somalia. While the authors acknowledge deficiencies in the quality and comparability of the included studies, they concluded that there is insufficient evidence to suggest that conflict increases the epidemic spread of HIV, at least in these geographic regions.

HIV prevalence in urban areas in Rwanda, Burundi and Uganda seemed to decline after periods of conflict while the rural prevalence remained stable [6]. In Juba, the largest town in Southern Sudan the prevalence of HIV is known from studies of outpatients to be 3.0% in 1995 and 4.0% in 1998. This is far below the prevalence of neighboring sites such as Mboki, in the Central African Republic, where HIV prevalence was measured at 11%. Similarly, HIV prevalence in the Acholi district of northern Uganda fell despite ongoing conflict from 1993 to 2003 (27% to 11.3%) [6]. It is likely that the relationship between HIV and conflict is not a uniform one, and, given the unique character of each conflict, generalizations are prone to error.

### Post-Conflict States

The end of formal hostilities frequently does not automatically herald improvements in the health indices of a given population. Nations emerging from conflict frequently have persistent difficulty in addressing healthcare needs. The cessation of hostilities commonly results in the unemployment of scores of young, uneducated, and unskilled men from either regular or irregular armed forces. Given the lack of opportunity in the face of economic privation, crime often spikes in the immediate post-conflict period [9-11]. If these unemployed former combatants are allowed to re-organize, secondary conflicts and organized crime may develop [11]. The addition of peacekeepers to post-conflict settings can further complicate the geometry of HIV transmission.

As has been seen in many African countries emerging from conflict, refugees and displaced persons have preferentially sought out large cities to seek employment and shelter after repatriation [6,8]. The concentration of migrant populations into already overcrowded cities, with inadequate or damaged health infrastructure, creates the potential for increased transmission of communicable diseases including HIV [3,4,6] Additionally, the commonplace violence, displacement, starvation and fear typical of the conflict phase can destroy social networks and prevent the concentration of people, therefore reducing the frequency of circumstances under which individuals may be exposed to HIV. The restoration of these networks, in the post-conflict phase, coupled with persistent shortages in health care and employment can create a fertile ground for HIV transmission.

It would seem that the period of recovery in the post-conflict phase is potentially a worrisome time for HIV transmission. Data is lacking and further study is required to better characterize this relationship. A careful analysis is required of the underlying determinants of HIV infection and subsequent AIDS-related mortality in conflict and post-conflict societies.

### Armed Parties

At the end of the Cold War in the 1990s, the nature of conflict changed as intra-state civil war became more prevalent than conflict between states. These new conflicts predominantly and asymmetrically affect the poorest of nations of the world and often the poorest populations within those nations. This change also reflects a shift away from conflict involving regular, uniformed forces to conflicts among and between rebel and insurgent groups and national armies [4,10,12]. These internal struggles have required substantial re-engineering of peacekeeping missions. In particular, recent peace operations have been large (tens of thousands of peacekeepers) and have increasingly employed peacekeepers from areas of relatively high underlying prevalence (e.g. the ECOWAS force in Liberia). Each of these armed populations represent unique and poorly studied variables that are likely to modulate transmission of HIV.

### Regular Military Forces

Soldiers have long been considered a high-risk population for HIV/AIDS. Indeed, initial data suggested that the prevalence of HIV amongst militaries was far in excess of the general populations in their home countries [2,3,12,13]. Multiple risk factors for HIV infection have been attributed to soldiers, including frequent commercial sex, risk taking mentality, concomitant sexually transmitted infection (STIs) and increasingly, injection drug use [1,2,4,8,10,13-15]. During conflict these behaviors may be exacerbated by stress and potentially limited command oversight. The role of iatrogenic infection via non-sterile injections, blood product transfusions, or medical procedures in the setting of a military medical system under combat stress have yet to be evaluated.

Soldiers are regularly sent to areas distant from their home and family support networks. In these settings soldiers, often the sole legal authority, are more likely to resort to commercial sex and/or coercive sex [4,8,14,15]. And soldiers in conflict regions may have more disposable income than the general population, further permitting commercial sex and risk taking behavior.

Recent data has suggested that the relationship between soldiers and HIV is not straightforward and studies have failed to demonstrate dramatically elevated HIV prevalence amongst military recruits. In 2000 the South African Defence Force (SADF) tested 10% of its active duty soldiers for HIV. A prevalence of 17% was found, which was not appreciably higher than among the general population [16]. Similar data has been found in Ethiopia where recruitment screening during mobilization in response to the war with Eritrea identified a relatively low seroprevalence of 2.8% [17]. These findings are attributed in part to demographic studies from South Africa and elsewhere demonstrating the relatively low HIV prevalence among the 17–22 year old age group (the age group from which recruits are drawn), as compared to older men and women [16]. Further, compulsory testing programs in many militaries, while problematic from a human rights standpoint, may allow national armed forces to at least initially select for an HIV-free population [18].

There is data to suggest that soldiers are at increased risk for contracting HIV, and that this risk increases with longer durations of service. Indeed, data from the SADF suggests an incidence of HIV infection of 1.2% per year of service [16]. Furthermore, data suggests that in the absence of unusual circumstances the HIV prevalence of a military unit will tend to stabilize to that of the population in which it is stationed, suggesting that the relatively low prevalence of newly recruited troops will not remain static [16]. It is unclear to what extent prevention and education campaigns can arrest this trend, and alternatively to what extent deployment for combat or peacekeeping may worsen this effect.

Demobilization after conflict is an additional concern. Victory, defeat, negotiated truce and/or the arrival of peacekeeping forces may herald the dissolution of all or part of the national military or insurgent forces. These armed, frequently uneducated, untrained and newly unemployed combatants often participate in criminal activity in the post-conflict period. Economic and societal factors may force these young men into cities to seek work, prolonging their isolation from family support networks and increasing their vulnerability to HIV infection. Demobilization of irregular forces in South Africa has been linked withthe spread of HIV, and a similar trend was seen in Cuban soldiers returning home after tours of duty in the Angolan conflict [16].

Multiple prevention initiatives have been adopted by the world's armed forces. A survey of militaries across the globe published in 2000, yielded the following statistics: 98% of militaries provided some form of HIV prevention education, 58% provided mandatory testing of all recruits and 17% turned away positive recruits [19]. Much research has been generated regarding HIV infection in militaries. Unfortunately, the majority of this data pertains to the militaries of the developed world [20]. Higher rates of HIV infection, illiteracy, and differing cultural and societal norms in many of the militaries of sub-Saharan Africa render extrapolation of such data difficult.

Some sub-Saharan countries have developed individualized HIV prevention strategies for their armed services. In Malawi, military recruits receive extensive counseling and education regarding HIV/STD infection and condom use [21]. Uganda has sought to de-stigmatize HIV infection and thus HIV testing by providing care and treatment for HIV positive service-members while protecting their rights and employment. The armed forces of Zimbabwe, Malawi and Zambia have instituted similar programs [21].

While the utility of many of these approaches remains untested, there is data to suggest a beneficial effect. A program piloted on Nigerian military personnel demonstrated that a "situationally focused" approach detailing avoidance of high-risk behaviors and situations could have beneficial effect on condom use and risk behaviors. At six months, risk behavior reporting decreased by 30% and by 23% at 12 months. Report of condom use increased significantly at both time points as well in comparison to baseline [22].

Other interventions, such as universal condom distribution to armed forces have encountered cultural and religious barriers, but may hold promise in preventing transmission. Data indicates that while the majority of armed forces provide recommendations regarding condom use, very few actually provide condoms to their soldiers [23]. Furthermore, recent data suggests a high prevalence of risk taking behavior on the part of soldiers in the post-deployment phase as they rejoin their families and social networks [23]. As well, given the experience in southern Africa regarding demobilization and HIV, post-deployment interventions may be an important component of HIV prevention strategies [16]. However, while a majority of services offer pre-deployment counseling and education to their troops very few offer post-deployment prevention education [23].

### Peacekeepers

Recent focus on peacekeeping has emphasized equipping, training and utilizing African forces in African peacekeeping operations. As discussed, soldiers display a multitude of risk behaviors potentially placing them at elevated risk for HIV infection. Nigerian peacekeepers returning to their home communities after operations in West Africa had rates of infection more than double that of the country overall [24]. There also appeared to be a dose response relationship, with the rate of infection correlating directly with the amount of time spent peacekeeping [24]. Incidence increased from 7% amongst troops peacekeeping for one year to 10% after two years and 15% after three years of deployment [24].

Similar to combatants in conflict zones, peacekeepers have been documented to engage in high-risk behavior while participating in missions [10,15]. While it is assumed that peacekeepers have access to healthcare, including treatment of sexually transmitted infections and HIV Voluntary Counseling and Testing (VCT), their sexual partners, including commercial sex workers, may not have access to these same resources. The impact of injection drug use on the transmission of HIV amongst peacekeepers during deployment has yet to be fully studied.

Several initiatives aimed at reducing HIV infection have been developed for soldiers participating in peacekeeping operations. The Department of Peacekeeping Operations (DPKO) and UNAIDS have developed and distributed an HIV/AIDS awareness card (with condom pocket) to peacekeepers [10,15,16]. This card has been translated into 15 languages spoken in 90 of the troop contributing nations. UNAIDS has also developed a programming guide, pre-deployment 'Standardized Generic Training Modules' and peer education kits for HIV education and prevention in troop contributing forces [10,15,16]. The DPKO endorses voluntary counseling and testing (VCT), as well as the availability of post-exposure prophylaxis (PEP) for peacekeepers [15,16]. Furthermore, as a result of a cooperative agreement between UNAIDS and DPKO, an AIDS advisor is in place with each of the current 16 peacekeeping missions [16].

### Insurgent Groups

Very little is known about the role of irregular troops in the spread of HIV. It can be argued that as these forces are frequently under inadequate command oversight and have access to limited medical support, they are potentially at higher risk than the soldiers of regular and peacekeeping forces. However, modern African insurgent groups are as diverse as the causes for which they fight, precluding ready generalization.

More so than in regular military forces, demobilization of insurgent groups is often incomplete, yielding persistent conflict despite any organized truce or cease-fire [25]. Further, even those who are demobilized may be incompletely incorporated into post-conflict society, remaining as marginalized populations or continuing to fight in criminal or insurgent groups. The dynamics of these relationships remain unknown and there is clear need for research in this area.

### Refugees/Internally Displaced Persons

Conflict and war often entails displacement of large groups both within and across national borders. These populations are frequently in crisis with their healthcare, nutritional, safety and shelter needs. Further, while countries are responsible for the care of individuals seeking safe haven on their soil, refugees have persistently been excluded from the planning and implementation of national HIV prevention, testing and treatment programs [4,26,27]. Given these factors one could assume that refugee groups would therefore have HIV rates far in excess of their host population.

This assumption has not been borne out by recent data. Spiegel et al examined HIV prevalence in refugee groups in comparison to their host communities [6]. Refugee populations were not found to have HIV prevalence in excess of the general populations of their hosts, and in many cases were significantly less infected, undermining the contention that refugee groups bring high rates of HIV infection to their hosts. For instance, refugees from the Democratic Republic of the Congo seeking refuge in the Gihembe camp of Rwanda had measured HIV prevalence of 1.5%, while the surrounding community (Byumba) had a prevalence of 6.7% [6]. Similarly, Sudanese refugees in the Kakuma camp in Kenya had HIV prevalence measured at 5%, while the surrounding community (Lodwar) demonstrated an HIV prevalence of 18% [6].

The effect of displacement on refugee populations could not be assessed due to the lack of reliable studies comparing pre and post displacement prevalence. However, there was a trend towards refugee groups slowly assuming the prevalence of their host population, suggesting that the final outcome is increased HIV prevalence amongst refugee groups in sub-Saharan Africa. It seems the majority of refugees in sub-Saharan Africa have fled from areas of low prevalence into areas of higher prevalence [6]. This finding points to another axis along which refugees – who have historically been viewed as vectors – might better be viewed as 'victims'. As with soldiers and peacekeepers returning to their home communities, there may be risk from repatriation of previously low prevalence refugee populations who have fled to areas of higher prevalence.

### Security Considerations

The interplay between HIV and conflict poses serious challenges to the nations of sub-Saharan Africa. Security has traditionally been thought of as pertaining exclusively to relationships between states [13,28,29]. Recently, however, thinking about security has evolved to include threats against the health and economic wellbeing of states. Indeed, the concepts of "collective security" or "biological security", as termed by former UN Secretary General Kofi Annan, demands a consideration of the health and well being of international populations [30].

There exists little evidence to suggest that HIV is a threat to the security of states in the traditional sense. However, through forcing the redirection of funds from development projects to HIV/AIDS care and via debilitating the labor forces, HIV is altering the trajectory of development and progress within many nations. Indeed, HIV/AIDS has significantly lowered the life expectancy across sub-Saharan Africa, reversing what had been decades of progress and creating massive disparities in life expectancy between some sub Saharan nations and the rest of the world [23,30].

In 2000 the UN Security Council addressed the notion of HIV as a threat to the security of nations. It was the first time a health issue had been the subject of a UN Security Council session [31]. The session noted that the HIV epidemic has, in many sub-Saharan countries, reversed decades of economic and social progress, and threatens substantial portions of the labor force as well as the economically active populace in multiple nations [10,31].

HIV also indirectly impacts national governments, as funds destined for social programs, development or security are reallocated to care for those infected and dying from HIV-related problems. Economic limitations related to the aftermath of conflict augmented by the cost of HIV/AIDS related spending, and loss of tax revenue related to increased mortality, may all profoundly limit medical and social investment. Additionally, as nations transition out of conflict, military populations with high HIV prevalence are demobilized and the fragile social balance achieved by cessation of hostilities may be jeopardized by the progression of the epidemic. National governments weakened by conflict may not be able to simultaneously support and fund reconstruction while dealing with a burgeoning HIV epidemic. As such, the ability of nations to move from conflict to post-conflict states, and to support and care for their populaces, may be constrained [10].

Lastly, in the absence of aggressive screening and prevention efforts, HIV has the potential to negatively impact the readiness and effectiveness of national armed forces. As soldiers become ill, funds and resources destined for equipping and arming the military and security forces may be reallocated to care for infected soldiers. For instance estimates from Kenya indicate that at the main military hospital 50–60% of inpatient hospital beds are occupied by HIV infected soldiers [32]. While concrete examples of security failure because of impaired readiness are lacking, it is certainly feasible that, in regions of high HIV prevalence, HIV/AIDS may negatively impact the ability of the armed forces to provide security in the face of combat stress.

### For the Future: Research and Programming Directions

In the above discussion several areas of need are clearly identified. We currently do not have substantial data regarding the effect of population displacement on HIV transmission. We can of course speculate that HIV prevalence increases in these settings, especially when refugees flee from areas of low HIV prevalence to areas of higher prevalence, or from rural to more urban areas. However, as we have learned with the conflict and HIV discussion, speculation is often done in error.

Data regarding post-conflict situations and the challenges inherent to this unique situation is lacking. Injection drug use is growing in sub-Saharan Africa, disproportionally so in conflict and post-conflict regions, yet little data exists describing this trend [33,34].

Research amongst displaced populations or in conflict and post-conflict settings is rife with difficulty and future studies must address the numerous biases and operational difficulties inherent in this work. Until adequate data is obtained it will be difficult to formulate programming interventions regarding these specific issues.

Further work must characterize the current approaches to HIV education, prevention and treatment among the militaries of the world, especially those of sub-Saharan Africa. Although military recruits may not have rates of infection far in excess of the general population, it is likely that they are at increased risk for HIV infection once deployed though it is not clear the extent to which conflict exacerbates this problem. Moreover, insurgent groups, often extremely marginalized have not been adequately studied, and data describing their role in the epidemic is lacking.

Lastly, it is of vital importance to continue to monitor the progression of the HIV epidemic in peacekeeping and security forces both in this region and globally. And critically this effort should not cease with demobilization.

## Conclusion

Recent data and discussion have caused reconsideration of many long held assumptions regarding the complex relationships between HIV, conflict and security. As such, previous generalizations must give way to a paradigm which recognizes the complexity inherent in these relationships and seeks to understand individual crises in their specific context. The data regarding HIV, conflict and security is incomplete and further investigation is required.

Nevertheless, several constants can be endorsed: the HIV epidemic poses severe challenges to the populations of sub-Saharan Africa. Nations in this region must be pro-active in addressing the epidemic amongst both the general population as well as the security and irregular forces. Failure to address these issues could hamper the ability of nations in this region to respond to crises, and as well threaten development efforts and the reconstruction and recovery that is vital in the post-conflict phase.

Numerous prevention and treatment efforts are underway among the militaries of the world, but data on this is lacking. While the effect of conflict and HIV on civilian populations is discussed, a parallel investigation into the effect of conflict on HIV in militaries should be widened.

The interaction between HIV, conflict and security is neither uniform nor straightforward. Nor is it likely to be stable. A tailored, coherent and thoughtful approach to these issues is required to inform policy and intervention regarding these dynamic relationships.

## Competing interests

There was no funding source for this publication other than the salaries of the three authors which are provided by their institutions (Yale University, University of Chicago). The authors attest that no other article or publication substantially similar in content to this has been published or is currently being considered for publication. There is no further conflict of interest or financial arrangement to be declared. No graphs, tables, or other media requiring release or permission is included in this manuscript.

## Authors' contributions

All authors certify sufficient participation in the conception, design, analysis, interpretation, writing, revising, and approval of the manuscript.
